# Supplementary material for: Different Polymers for the Base of Removable Dentures? Part II: A Narrative Review of the Dynamics of Microbial Plaque Formation on Dentures
Source: Polymers (Basel). 2023 Dec 21;16(1):40. doi: 10.3390/polym16010040 (PMC10780608; doi:10.3390/polym16010040)
Supplement: Supplementary file 1 [file polymers-16-00040-s001.zip › polymers-2508065-supplementary.pdf]

Table S1. In vitro coating or addition of antimicrobial components in PMMA.

PMMA	Antimicrobial nanoparticules 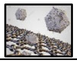	findings	Results
Natural Resin, Nisshin Co, Kyoto, Japan.	Adding 1, 5, 10 wt% of apatite-coated titanium dioxide (Ap-TiO2) to acrylic resin was expected to produce antifungal effects.	It was found that excessive amount of Ap-TiO2 should be avoided because of its detrimental influence on mechanical properties.	Acrylic resin containing 5 wt**%** of Ap-TiO2 could exert antifungal effect on *C. albicans*, while at the same time maintain adequate mechanical properties for clinical use [197].
A microwave-polymerised (Onda-Cryl)<br>two heat-polymerised : (QC20 ; Lucitone 550) acrylic resins	The antimicrobial activity against two strains of *Candida albicans* and two strains of Streptococcus mutans was assessed by agar diffusion method. The addition of 0,2.5, 5,7,5, 10 % of Irgaguard B5000 (silver-zinc zeolite) to the materials tested in antimicrobial activity against all strains.	-Flexural strength decreased significantly with the addition of 2.5% (QC20 and Lucitone 550) and 5.0% (Onda-Cryl) of Irgaguard B5000.<br>-The impact strength decreased significantly with the addition of 2.5% (Lucitone 550) and 5.0% (QC20 and Onda-Cryl) of zeolite.	The addition of silver-zinc zeolite to arylic resins yields antimicrobial activity, but may affect negatively the mechanical properties [198].
Thermopolymerized acrylic resin, Nature-Cryl™ (GC America Inc, Alsip, IL) (control).	Pink PMMA powder was synthetized using the suspension polymerization technique, adding TiO_2 and Fe_2O_3 nanoparticules . The PMMA monomer was added with 1 µl/ml of silver nanoparticules.<br>The antifungal effect was assessed using a luminescent microbial cell viability assay.	-PMMA-silver nanoparticle discs significantly reduce adherence of C. albicans and do not affect metabolism or proliferation. They also appear not to cause genotoxic damage to cells.<br>The flexural properties of the PMMA-silver nanoparticules show that the main values were according to the ISO – 1567.	Silver nanoparticles are biocompatible antifungals incorporated into PMMA bases for dental prostheses [199].
Lucitone 550 denture resin	Silver nanoparticle suspension was added to the acrylic resin monomer in different concentrations (0.05, 0.5, and 5 vol% silver colloidal).<br>The specimens were stored in deionized water at 37°C for 7, 15, 30, 60, and 120 days, and each solution was analyzed using atomic absorption spectroscopy.	Incorporation of silver nanoparticules added to the resin and after 120 days of storage were mainly located on the surface of the nanocomposite specimens.	Silver nanoparticles are incorporated into PMMA prosthetic resin to achieve an effective antimicrobial effect during 120 days to control common infections involving oral mucosal tissues in complete denture wearers [200].
Acrylic resin	Adding three types of TiO2 powder [FAp-TiO2, titanium dioxide (TiO2) and hydroxyapatite-coated TiO2 (HAp-TiO2).	Fluoridated apatite-coated titanium dioxide-containing acrylic resin demonstrated superior effectiveness in inhibiting C. albicans adherence as well as in decomposing MB.	Acrylic resin coated with fluorinated apatite containing titanium dioxide, is a clinically suitable material that promotes hygiene [142].
Heat polymerized PMMA powder (Urban, clear pink, C2 , Shofu Inc, Kyoto Japan) with monomer (Shofu Inc, Kyoto Japan) .	Adding, four concentrations of fluoridated glass: 1%, 2.5%, 5% and 10% by weight pre-polymerization and 0% was the control.<br>Specimens were incubated with *Candida albicans, or Streptococcus mutans* with and without saliva coating. Adherence was presented as a percentage of the colonized surface area, counted using an optical microscope at x100 magnification.	The fluoridated glass fillers concentration 10% concentration significantly decreased candidal and bacterial adhesion compared to others. Saliva coating significantly decreased microbial adhesion.<br><br>The metabolic activity and total biomass volume were significantly lower in all filler groups than in the control group.	Fluoridated glass fillers could decrease microbial adhesion to acrylic denture base without adversely affecting surface properties [201].

PMMA-based, heat polymerizing resin	Discs were prepared by incorporating the surface reaction-type prereacted glass ionomer (S-PRG) filler at 0 (control), 5%, 10%, and 20% (w/w)<br><br>Each disc was placed artificial saliva and immersed in a *C. albicans* cell suspension standardized at 10(4) cells/ml. After aerobic incubation at 37 °C for 24 h, the metabolic mitochondrial activity, total biofilm biomass, and biofilm thickness were evaluated. The morphogenetic transition of C. albicans in the early culture stage (1 and 3 h) was observed.	- All filler groups showed hyphal Candida forms at 3 h, with the length of the hyphae being lesser than those in the control group.<br>-Although the incorporation of S-PRG filler slightly increases the surface roughness of denture base resin, it reduces the adhesion of C. albicans.<br>-The S-PRG filler has the potential to reduce Candida albicans adhesion on denture base resin and may lower the risk of denture stomatitis.	Groups with at least 5% filler content exhibited a thinner biofilm compared with the control group [202].
PMMA denture base material	Add silver nano-particles (Ag NPs) at concentration 1, 1,5, 2, 2,5 w%.<br>The effect of silver NPs on the reaction kinetics is investigated by measuring the variation of conversion with time and the molecular weight distribution of the polymer formed.	AgNP agglomerates show a slight decrease of the antimicrobial effect, which is observed more at higher concentrations (2 wt %) compared with lower concentrations (1.5 wt %), and well-dispersed nanoparticles. The authors conducted the antimicrobial tests against *P. aeruginosa* and *S. aureus* and they studied the antifungal effectt against *Cryptococcus neoformans*. In all cases, concentrations lower than 0.75 wt % had no well-defined inhibition zone when observed in agar plates.	When using concentrations between 1 and 1.5 wt %, authors observed positive results against Gram-negative and Gram-positive bacteria and an antifungal effect [203].
PMMA denture base material	Mesoporus silica nanoparticules MSN-incorporated in PMMA: 0,5, 1, 2,5 or 5 wt %	-An anti-adherent effect against *Candida albicans* and *Streptococcus oralis* after 1h of attachment was only observed with 2.5 and 5wt% incorporation compared to a lack of MSNs.<br>-A long-term antimicrobial effect was observed for 2 weeks with 2.5wt% MSN-incorporated PMMA when amphotericin B was loaded into the MSNs on the PMMA surface.	The long-term antimicrobial performance after loading amphotericin B into the MSN-incorporated PMMA suggests the potential clinical usefulness of MSN-incorporated PMMA resin [204].
PMMA denture base material	Silver microparticles were added to the polymer powder in different concentrations by volume (0%, 0.25%, 0.5%, and 1%), antifungal activity against Candida albicans was assessed in terms of colony-forming units. PMMA disc specimens containing silver microparticles were eluted with culture medium for 1, 2, and 5 days. The cytotoxicity of the eluates to cultured L929 assay.	-The antifungal effect against *C. albicans* increased with the percentage of silver microparticles.<br>-For both tests, both RTCA and the MTT assay, no time- or silver-dependent cytotoxicity of PMMA denture base material containing silver microparticles was observed.	PMMA denture base material containing silver microparticles have antifungal activity and no cytotoxic effect [205].
Cold polymerized acrylic resin-PMMA and MMA (Triplex Cold, Ivoclar Vivadent, Liechtenstein),	Oleic acid (OA), is a natural compound, preventing filamentation (transition from the yeast state to filamentation). The incorporation of 3%, 6%, 9% and 12% OA into PMMA was tested for 0 and 6 days.<br>mouse fibroblast cells was evaluated using a RTCA system and the MTT	In vitro, OA considerably reduces the contact angle with water. It also helps reduce the metabolic activity of biofilm and candidal planktonic cells.	PMMA-OA composites with 3% OA significantly reduces metabolic activity of biofilm cells even 6 days [206].

The effects of the incorporation of different nanoparticles (Ag NPs, silver-zinc zeolite, TiO_2 and Fe_2O_3) (Fluoridated glass fillers) (the surface reaction-type prereacted glass ionomer (S-PRG)) (Mesoporus silica nanoparticules) or Oleic acid (OA) like a natural compound, in PMMA can produce antifungal and antibacterial effects, limiting the development of biofilm on the surface of the prosthesis. However, if the dosage of these components is not respected, harmful mechanical effects are observed as well as undesirable cytotoxic effects.

Table S2. Cytotoxicity and biocompatibility of PMMA, Polyamide.

**Polymers**	**Cytotoxic test** 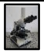	**findings**	**Reference**
- PMMA heat-cured processed with conventional pressure-pack technique (QC20)<br>- PMMA self-cured polymerized at room temperature (SC Cold Cure)<br>- Polyamide resin Deflex specimens plasticized at 270C in injection flasks	Direct cell contact method using cell viability and neutral red (NR). tested at initial and after being aged for 24 h, 1 week and 8 weeks with artificial saliva according to ISO 10993 standards.	-After 24 hours, Deflex showed higher toxicity than the control group ($p < 0.05$).<br>-After one week, all materials reached their peak values, with no statistically significant difference compared to the initial and 24-hour cell viabilities.<br>-After eight weeks, all materials were more toxic than the control group, as well as the initial, 24-hour, and one-week aging times ($p < 0.05$).<br>-Among the materials, QC-20 exhibited the highest toxicity after eight weeks, significantly differing from Deflex and SCCold Cure ($p < 0.05$).<br>-Polyamide displayed a toxicity profile like conventional Polymethyl methacrylate denture base materials in all tests..	**Polyamide displayed a toxicity profile like conventional PMMA denture in all tests.**<br>**[207].**
- PMMA heat polymerized (Lucitone 199, Dentsply)<br>- Polyamide nylon 6 polyamide (ValplastTM, Valplast International)<br>- Polyamide nylon 12 composite polyamide (DuraflexTM, Myerson).<br><br>(polished disks/ unpolished disks)	Culture gingival epithelial cells and fibroblasts conditioned media prepared from denture material disks and then assayed cell toxicity by MTT cell viability assay.	-The three denture materials exhibited varying levels of toxicity towards the cells. Valplast conditioned media, particularly those from unpolished disks, demonstrated significant toxicity to both gingival fibroblasts and epithelial cells. In contrast, media obtained from polished Lucitone and Duraflex showed comparatively lower levels of toxicity.<br>-Following a 7-day incubation period with Valplast unpolished conditioned media, only 1 to 2% of the cells remained viable. In contrast, the polished disk conditioned media caused significantly less toxicity ($p < 0.05$), with approximately 76% and 92% viability observed for fibroblasts and epithelial cells, respectively.<br>-When incubated with media derived from the other denture materials, cell viability ranged from 35% to 92% for both fibroblasts and epithelial cells.<br>-It is worth noting that the data obtained from the LDH assay and the live/dead mammalian cell viability assay were consistent with the results obtained from the MTT viability assay.	Concerning polyamide (Valplast) the level of polishing influences its toxicity<br>**[208].**
- PMMA Paladent 20 (Heraeus Kulzer, Hanau, Germany/ Compression molding)<br>- Polyamide Bio Tone (High–Dental-Japan, Osaka, Japan/ Injection molding)<br>- PMMA Acrytone (High–Dental-Japan, Osaka, Japan/ Injection molding)	Cell Human gingival fibroblasts viability assay was measured EZ-Cytox Kit (Daeillab service co., Seoul, South Korea) and cell attachment was analyzed by FE-SEM (HITACHI S-4800, Tokyo, Japan).	-After 1 day, cell viability remained unaffected in both the specimens and the control group, with increased viability observed in the polyamide group. However, the PMMA-based resin exhibited reduced absorbance and lower cell viability compared to polyamides on the 6th day.<br>-On day 1, Bio Tone exhibited the smoothest pre-test surface and the most efficient cell attachment, while Acrytone and Paladent 20 showed moderate and poor cell attachment, respectively.<br>-By day 6, Bio Tone had the highest hGF cell attachment. On day 10, Bio Tone displayed the most abundant cell attachment, while Acrytone and Paladent 20 showed similar cell attachment levels, although Acrytone demonstrated a more stable pattern.<br>-The cytotoxicity of thermoplastic acrylic resins resembled that of thermoplastic polyamides and conventional heat-polymerized acrylic resin.	There is little difference between the cytotoxicity of thermoplastic acrylic resins, that of thermoplastic polyamides and thermopolymerized acrylic resins **[209].**
PMMA : Heat cure acrylic resin (vertex – Dental B.V, Zeist, Netherlands) polymerized using conventional compression flask technique.<br>Polyamide : Thermoplastic acrylic resin discs (Bre. flex polyamide, Bredent, Gmbh. Co.K.G. Senden, Germany) injection molding technique.	Cytotoxicity of hMSCs (Lonza, Germany) was evaluated by WST-1 assay (Roche applied science, Germany) and Live/dead fluorescent cell viability	-After 24 hours, both groups exhibited a higher survival cell rate compared to the control group, with a slightly higher rate of surviving hMSCs in PMMA, although this difference lacked statistical significance.<br>-Green, fluorescent cells were observed in both PMMA and PA, with fewer scattered red fluorescent cells in PA. However, the differences in fluorescence patterns between the two groups were not statistically significant.<br>-The polymerization method employed in both groups did not impact the cytotoxicity or biocompatibility of the denture base resins.	There is no difference between PMMA and polyamide.<br>**[210].**

	staining (Calcein-AM stains while Ethidium homodimer III (EthD-III)<br>Control group : hMSCs cultured in complete culture media for 24 h. Idem for PMMA, PA .		
Three Polyamides resin-based products:<br>1- Smile tone (ST, Korea Engineering Plastics Co., Seoul, Korea),<br>2-Valplast (VP, Valplast) (USA)<br>3-Luciton FRS (LF, Dentsply Trubyte); (USA)<br>Two thermoplastic acrylic resin-based :<br>1-Acrytone (AT, High Dental) (Japan)<br>2- Acryshot (AS, High Dental) (Japan)<br>One Polypropylene-based :<br>Unigum (UG, Weldenz) (Japan)<br>One conventional heat-polymerized acrylic resinbased:<br>Vertex RS (RS, Vertex) (Netherlands)	All specimens of the denture resin materials under extraction condition (37°C, 70°C 121°C) in distilled water during 24H were co cultured with human oral keratinocytes (IHOKs) or mouse fibroblasts (L929s).	-Severe toxicity (less than 70%) was not detected in any tested thermoplastic denture resin used in non metal clasp dentures when human oral keratinocytes IHOKs and L929s were subjected after incubation at different temperatures .<br>Lower IHOK and L929 viability was observed with the 50% extract from VP (70°C) and AT (121°C) samples ($P<.05$). L929 alone exhibited reduced viability with the 50% and 25% extract from LF (37°C) ($P<.05$).<br>-Extracts from six materials, across varying extraction conditions (37°C, 70°C, and 121°C), did not display significant cytotoxicity (viability > 70%). However, their potential impact on oral mucosa at elevated temperatures should not be overlooked.	The potential risk to oral mucosa at high temperature should not be ignored for the six materials.<br>**[211].**
-Heat cured acrylic<br>-Flexible resin : addition of silver vanadate nanorods / addition of titania nanorods.	Cell toxicity: Tryptan-blue staining after 24 and 48 h of incubation of the discs with cells.	-Silver nanorods adversely affect the cytotoxicity of both flexible and heat-cured acrylic resins.<br>-Titania nanorods are biocompatible materials and do not induce cytotoxicity when used with flexible resin.<br>-The incorporation of titania nanorods increases the impact strength of both flexible and heat-cured acrylic resins.<br>-Flexible resin exhibits lower cytotoxicity compared to heat-cured acrylic resin.<br>-Titania nanorods enhance the impact strength and surface roughness of flexible resin when compared to heat-cured acrylic resin..	**[212].**
Cytotoxic effects of heat and auto-polymerized acrylic resin:<br>1-Vertex (V) Vertex-dental, Zeist, The Netherlands.<br>2- Orthocryl (O),Dentarium, Ispringen, Germany.<br>3- Imident (I),Imicryl, Konya, Turkey.<br>4-Paladent (P) ,Ped-dent , Ankara, Turkey.<br>5- Meliodent (M) Heraeus Kulzer, hanau, Germany.<br>-Particulate filler resin composite :<br>6-Signum (S),Heraeus Kulzer, Hanau, Germany.<br>7-Adoro (A), Ivoclar-vivadent , Schaan, Liechtenstein.<br>8-Tescera (T) Bisco, Schaumburg, IL, USA.<br>-Thermoplastic material:<br>9-Bioplast (B) AmBurgberg, Iserborn, Germany.	. All samples were divided into two groups to be preserved either in artificial saliva (AS) or with melatonin (ASM). - After the incubation of the samples in (AS) and (ASM) the incorporation of mouse L-929 fibroblast cells for 1, 24, 72 h, 1 week and 2 weeks made it possible to test MTT cell viability.	-Cytotoxicity results showed no signifiant difference between the AS and ASM .<br>- L'incubation (L-929 fibroblast) significantly affected all materials tested.<br>-There was no significant difference in cytotoxicity between the O, V, and I materials in the ASM after a 1-hour incubation period. However, the absorbent index values increased for the M, A, T, B, S, and P materials during this timeframe.<br>-Specifically, auto-polymerized acrylic resin specimens (M) exhibited no change in cytotoxicity levels after 1 hour. In contrast, heat-polymerized particulate filler composite resins and thermoplastic materials showed a decrease in cytotoxicity after 1 hour of incubation.<br>- After 24 h, storing resin-based materials in (ASM) reduce cytotoxicity effects of fibroblast cells for which the highest effect was observed.	Soaking resin prosthesis in artificial saliva with melatonin before intraoral use may be recommended for decreasing the cytoxicity of dental resin materials<br>**[213].**

The *in vitro* comparison between PMMA and polyamide regarding cytotoxicity does not reveal obvious differences. Studies remain disparate with regard to the materials studied and the protocols used. The results fluctuate depending on the duration of the experiments and different parameters such as temperature and surface condition.
